# Temperature Rise Increases the Bioavailability of Marine *Synechococcus*-Derived Dissolved Organic Matter

**DOI:** 10.3389/fmicb.2022.838707

**Published:** 2022-04-19

**Authors:** Jiajie Zhang, Jihua Liu, Daixi Liu, Xiao Chen, Quan Shi, Chen He, Gang Li

**Affiliations:** ^1^Institute of Marine Science and Technology, Shandong University, Qingdao, China; ^2^Joint Lab for Ocean Research and Education at Dalhousie University, Shandong University and Xiamen University, Qingdao, China; ^3^Southern Marine Science and Engineering Guangdong Laboratory, Zhuhai, China; ^4^State Key Laboratory of Heavy Oil Processing, China University of Petroleum, Beijing, China; ^5^Key Laboratory of Tropical Marine Bio-resources and Ecology, South China Sea Institute of Oceanology, Chinese Academy of Sciences, Guangzhou, China

**Keywords:** dissolved organic matter (DOM), bioavailability, temperature rise, heterotrophic bacteria, exponential and decay phases, *Synechococcus* sp. PCC7002

## Abstract

*Synechococcus* is one group of main primary producers and plays a key role in oceanic carbon fixation and transformation. To explore how the temperature rise affects the bioavailability of *Synechococcus*-derived dissolved organic matter (SOM) and whether this effect would be altered by the involvement of heterotrophic bacteria, we compared the optical and molecular properties of the SOM of axenic *Synechococcus* sp. PCC7002 culture (*Syn*) to that with associated heterotrophic bacteria (*Syn*B) under 15, 18, and 21^°^C growth temperatures at exponential and decay growth phases. Our results showed that the temperature rise increased the bioavailability of the SOM of both *Syn* and *Syn*B cultures by lowering the proportion of the hydrogen-poor and double-bond structure-rich humus-like components and highly unsaturated substances, as indicated by the increase of spectral slope ratio (S_*R*_) and biological index (BIX) and decrease of humification index (HIX). Moreover, the involvement of heterotrophic bacteria modified the *Synechococcus*-derived SOM, together with its intracellular dissolved organic matter (DOM) excludes, lowering the SOM bioavailability. Our results indicated that the warming in climate change scenario may enhance the bioavailability of the *Synechococcus*-derived SOM although it may be tempered by the involvement of heterotrophic bacteria, providing an insight for preservation of the organic carbon pool in global oceans.

## Introduction

*Synecchococcus* is a significant group of photosynthetic carbon sequestration organisms that are widely distributed in coastal and oceanic environments, and is thus of great value in marine ecosystem (Huang et al., 2011; Jiao et al., 2011). Previous studies predicted that ocean warming will promote the importance of *Synechococcus* in the oceanic carbon cycle through expanding their distribution range and increasing their cell abundance (Morán et al., 2010; Flombaum et al., 2013). The *Synechococcus*-derived dissolved organic matter (SOM) that is released into surroundings via secretion, natural cell death, viral lysis, and predation (Jiao et al., 2010; Fiore et al., 2015; Xiao et al., 2020) largely contributes to the marine dissolved organic matter (DOM) pool (Jiao et al., 2011; Gontikaki et al., 2013), and is thus one of the main drivers for the oceanic carbon cycle (Dufresne et al., 2008). The ultimate fate of SOM is usually determined by the associated heterotrophic bacteria in the ocean, and the bioavailability of SOM influences its persistence and the development of DOM pools (Jiao et al., 2010; Sarmento et al., 2016). Generally, the bioavailability of DOM refers to the consumed or/and utilized degrees of DOM compounds by associated heterotrophic bacteria, which primarily depends on their chemical composition (Berggren and Giorgio, 2015). Contrary to common belief, the DOM produced by algae did not always have high bioavailability (Koch et al., 2014). For example, a study by Koch et al. (2014) showed that only 20% of organic carbon from marine algae was degraded during the 2-year cultivation. Moreover, the DOM induced by viral lysis from cyanobacteria was detected to have similar optical properties with that from natural deep-ocean, indicating a great possibility that the cyanobacteria-derived DOM directly contributes to the refractory DOM (RDOM) formation (Zhao et al., 2017).

Ocean warming is accelerating, with surface seawater temperature being expected to increase by 1–7^°^C by the end of this century (Kattsov et al., 2001; Levitus et al., 2005; Gleick et al., 2010; Cheng et al., 2019). Previous studies indicated that global warming enhances the bioavailability of marine DOM (Segschneider and Bendtsen, 2013). The available evidence also indicated the possible effects of ocean warming on the bioavailability of SOM. For instance, many studies reported the temperature rise deeply affected the physiology, biochemical compositions, and carbon fixation of *Synechococcus* (Fu et al., 2007; Paerl and Huisman, 2008; Flombaum et al., 2013; Harvey et al., 2013; Paul et al., 2018). Furthermore, the elevated temperature has been observed to accelerate the production of phytoplankton fluorescent DOM (FDOM) (Yang et al., 2020). The temperature rise was also observed to be tightly coupled with the amount of extracellular microcystin (Walls et al., 2018), heterocyst glycolipid ketone alcohols (Bauersachs et al., 2014), and fatty acid (Nalley et al., 2018) produced by cyanobacteria. However, it is unclear about the effects of temperature rise on the molecular properties of SOM.

The exudations of both healthy and dead *Synechococcus* cells contribute to the SOM within surroundings (Agustí et al., 1998; Marañón et al., 2004; López Sandoval et al., 2013; Becker et al., 2014). Approximately, 25–65% of environmental DOM is released by cell lyse, viral lysis, or grazing activity, and is considered as intracellular DOM (Hygum et al., 1997; Myklestad and Swift, 1998; Møller et al., 2003; Malits et al., 2021). Compared with the intracellular DOM released due to cell death, the extracellular DOM secreted during cell growing, e.g., chromophoric DOM (CDOM) and FDOM, were more photolabile, with faster photochemical removal (Bittar et al., 2015). In addition, the molecular weight, double bond equivalents (DBE), hydrogen to carbon ratio (H/C), and modified aromatic index (AImod) of the algae-associated DOM often increase with algae cell growing or decay durations, indicating the transformation from active to refractory DOM (Liu et al., 2021). Apart from that, heterotrophic bacteria also play an important role in both the growth of *Synechococcus* and transformation of SOM (Zheng et al., 2017, 2019). Experiments assessing the SOM without the effects of heterotrophic bacteria have so far been scarce. In return for utilization of the organic carbon fixed by cyanobacteria, the heterotrophic bacteria benefit them by providing complex and unidentified substances like vitamins and bioavailable trace metals (Amin et al., 2009; Kazamia et al., 2012). Because of this, rendering axenic cyanobacteria is difficult for laboratorial cultures. Therefore, few studies have so far focused on how temperature affects the bioavailability of DOM in *Synechococcus*, especially under different growth phases and with/without heterotrophic bacteria transformations.

It is of great significance to study the bioavailability change of SOM under elevated temperature when considering its contribution to the oceanic carbon pool. Cyanobacteria like *Synechococcus* are one group of the dominating photoautotrophic microorganisms in the middle and low latitudinal coastal waters (Flombaum et al., 2013), while the *Synechococcus* sp. PCC7002 is a model strain and mainly distributed in the coastal waters (Mou et al., 2017, 2018). Therefore, in this study we examined the changes of the properties of DOM from *Synechococcus* sp. PCC7002 with and without the associated heterotrophic bacteria effects under different growth temperatures (15, 18, and 21°C) at exponential and decay growth phases. This study intended to elucidate how the increased temperature influences the persistence of SOM pool in global oceans.

## Materials and Methods

### Experimental Design

The marine *Synechococcus* sp. PCC7002 culture with microbes was obtained from Xiamen University Center for Marine Phytoplankton. In this culture, 30 mainly associated bacteria strains belonging to six species (Supplementary Table 1) were identified with agar 2216 medium. Among them the *Halomonas* sp. that belongs to class *Gammaproteobacteria* was dominant and isolated with streak plate methods and used in the following experiments. The axenic *Synechococcus* sp. PCC7002 was isolated by streaking onto the A^+^ agar plate and purified by repeatedly culturing in solid and liquid media (Zhang et al., 2021).

To probe how heterotrophic bacteria affect the bioavailability of SOM, we cultured both axenic *Synechococcus* sp. PCC7002 (*Syn*) and that with *Halomonas* sp. (*Syn*B) in A^+^ medium with the Fe-EDTA, Tris-HCl, and vitamin B12 concentrations being adjusted to 7.5 μM, 4 mM, and 3 nM, respectively (Stevens and Porter, 1980; Ludwig and Bryant, 2011). Such an adjustment was to eliminate the influence of external organic carbon while maintaining the growth of *Synechococcus* sp. PCC7002. Both the *Syn* and *Syn*B cultures were grown in 300-ml conical flasks (50-ml cultures) under the temperatures of 15, 18, and 21^°^C in a light incubator (ZQZYCGF8, Shanghai, China). Continuous growth light was provided by a panel of LEDs (Light Emitting Diodes) on top of the incubator with light intensity of 20 ± 1.0 μmol photons m^–2^ s^–1^, that was measured with a microscopically quantum sensor (QRT1, Hansatech, United Kingdom) in a culture flask filled with medium. Such a low growth light was chosen to limit the photodegradation or photo transformation of the *Synechococcus*-derived SOM, as well as maintain the favorable growth of *Synechococcus* sp. PCC7002 (Mackey et al., 2017). Under each temperature, 20 replicate flasks for each *Syn* and *Syn*B culture were maintained for the subsequent determination of growth, SOM absorption, and fluorescence and molecular compositions. In each flask, the initial *Synechococcus* sp. PCC7002 cell density was adjusted to ∼10^7^ cells ml^–1^, and extra *Halomonas* sp. isolated above was co-inoculated in *Syn*B culture to the density of ∼10^6^ cells ml^–1^ (Zhang et al., 2021). Before inoculating to the *Syn* culture, the bacteria was pre-cultured 2 days in marine broth 2216 at 33°C and washed twice with the modified A^+^ medium. All the glassware used in this experiment was pre-combusted at 450^°^C for 5 h to eliminate the effects of external organic carbon.

### Growth Phase Determination

Growth of *Synechococcus* sp. PCC7002 in *Syn* and *Syn*B cultures was tracked through monitoring the changes in cell abundance using a flow cytometer (Accuri^§^ C6, Becton-Dickinson, Franklin Lakes, NJ, United States). Every 1–3 days, 1-ml culture was taken out from random three flasks of *Syn* and *Syn*B cultures under each temperature, and the cell abundance was measured. In addition, the decay phase can be primarily identified with the visible color changes, i.e., from blue-green to yellow-green.

We identified the exponential growth phase at Days 10–32 in *Syn* and at Days 14–35 in *Syn*B cultures following Mou et al. (2018), and calculated the specific growth rate (μ_*max*_, d^–1^) with the equation:


$$\mu_{\max}=\left[L\,N\,\left(N_{t}\right)-L\,N\,\left(N_{t\,0}\right)\right]/\left(t-t_{0}\right)$$

where N_*t*_ and N_*t*0_ are the culture cell abundance at the time t and t0, respectively.

### Dissolved Organic Matter Absorption and Fluorescence Measurements

In the exponential growth phase, random three replicate flasks of *Syn* and *Syn*B cultures were taken out from the incubator to measure DOM absorption and fluorescence. From each flask, 5 ml culture was taken out and filtrated through a pre-combusted (450^°^C, 5 h) GF/F filter (Whatman, 25 mm in diameter), and the filtration was collected, dispensed into a brown glass tube, and stored at 4°C after 10-time dilution until later measurements of absorption and fluorescence within 3 days. After sampling, these flasks were discarded to eradicate contamination. At the end of cultivation, such a sampling process was performed again, for the DOM determinations in decay growth phase.

To measure the DOM absorption, the filtration was put into a 1-cm quartz cuvette of spectrophotometer. The DOM absorption spectrum was scanned from 250 to 600 nm with a 1-nm increment, referencing to Milli-Q water. Mean absorptions from 575 to 600 nm were used to correct the effects of scattering and baseline fluctuation (Helms et al., 2008). The absorption coefficient of CDOM was calculated (Bricaud et al., 1981) as:


$$a_{C\,D\,O\,M}\,\left(\lambda \right)=2.303*A_{C\,D\,O\,M}\,\left(\lambda \right)/L$$

where a_*CDOM*_(λ) is the corrected CDOM absorption coefficient at wavelength λ, A_*CDOM*_ (λ) is the corrected optical density at wavelength λ, and L is the path length (m).

The spectral slope was calculated using a non-linear least squares fitting routine over the range of 275–295 nm (S_275–295_) and 350–400 nm (S_350–400_) (Nvdv, 2002) as:


$$a_{C\,D\,O\,M}\,\left(\lambda \right)=a_{C\,D\,O\,M}\,\left(\lambda \,0\right)\,e\,x\,p\,\left[S\,\left(\left(\lambda \,0-\lambda \right)\right)\right]$$

where λ*o* is a reference wavelength and S is the spectral slope parameter.

The spectral slope ratio (S_*R*_) was defined as the ratio of the spectral slope of 275–295 nm to that of 350–400 nm. Generally, the average molecular weight and aromaticity of CDOM are inversely proportional to S_*R*_: Lower S_*R*_ indicates higher aromaticity, while higher S_*R*_ indicates lower aromaticity and thus easier biological degradation (Helms et al., 2008).

To measure excitation emission matrix (EEM), the DOM sample was dispensed into a 1-cm quartz cuvette and measured using a fluorescence spectrophotometer (Cary Eclipse, Agilent, Santa Clara, CA, United States) with the excitation and emission wavelengths of 250–550 nm and 250–600 nm, respectively. The excitation and emission slit widths were set as 10 nm, with the scan speed of 1,200 nm min^–1^. Parallel factor analysis (PARAFAC) was performed in MATLAB 2021a with the drEEM toolbox 0.6.3 (Murphy et al., 2013). Moreover, the Milli-Q water was used as blank to calibrate the EEMs data (Lawaetz and Stedmon, 2009; Guo et al., 2011), and all the fluorescent data were presented in Raman Units (RU), from which the humification index (HIX) (Ohno, and Tsutomu., 2002), and biological index (BIX) (Huguet et al., 2009) were calculated.

### Molecular Signature Determinations

After measuring DOM absorption and fluorescence, the remaining cultures (∼40 ml in each of three replicate flasks) were mixed together, filtered through a GF/F filter, and collected into a pre-combusted glass bottle. The collected filtrate was extracted through methanol-rinsed and formic acid-acidified PPL solid-period extraction cartridges (500 mg, Agilent Technologies) with the speed of ∼5 ml min^–1^. After extraction, the cartridges were washed with 15 ml formic acid (Sigma-Aldrich, Darmstadt, Germany, 98%), dried, and stored at -20^°^C for later DOM molecular signature analysis with a Fourier transform ion cyclotron resonance mass spectrometry (FT-ICR MS). The DOM molecular signature analysis was conducted by a 9.4 Tesla Apex-ultra FT-ICR MS in the Heavy Oil Key Laboratory, School of Chemical Engineering, China University of Petroleum, Beijing, China (He et al., 2020). To determine molecular properties, the DOM in PPL was eluted with 10 ml chromatographically pure methanol (Sigma-Aldrich, Darmstadt, Germany) and injected into instrument at speed of 180 μl h^–1^. The operating conditions for negative formation were set as: 3.0 kV emitter voltage, 3.5 kV capillary column introduce voltage, and -320 V capillary column end voltage, and the samples were scanned over an *m/z* range of 200–800. Then, based on the AImod index (Qiao et al., 2020) and H/C, the DOM compounds were grouped into five classes (Seidel et al., 2014): polycyclic aromatics (AImod > 0.66), polyphenols (0.5 < AImod < 0.66), highly unsaturated and phenolic compounds (AImod ≤ 0.5 and H/C < 1.5), aliphatic compounds (1.5 ≤ H/C < 2), and saturated compounds (AImod < 0.5 and H/C > 2). The intensity of molecular formula is also taken into account when calculating the percentage of each class. The DBE values were calculated (Koch and Dittmar, 2006) to characterize the number of double bonds in molecules.

### Statistical Analyses

All the parameters were presented with mean and standard deviations (mean ± SD) of three independent biological replicate cultures except for the FT-ICR MS data from a mixture of the three replicates. Paired *t*-test was performed with OriginPro 2021 software (Origin Lab Corporation, Northampton, MA, United States), and a linear regression was applied to test the correlation of measured parameters to temperature. The significance level was set as *p* = 0.05.

## Results and Discussion

### *Synechococcus* Growth

**FIGURE 1:**
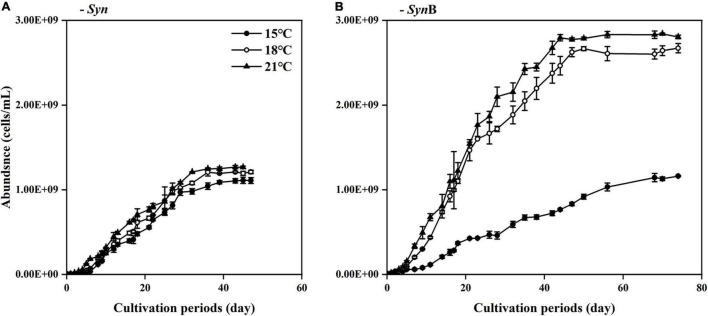
Changes of cell density (indicated by adjusted OD_730_) of *Synechococcus* sp. PCC7002 in cultures without [**(A)**, *Syn*] and with heterotrophic bacteria [**(B)**, *Syn*B] during cultivation periods under temperatures of 15, 18, and 21^°^C. Points show averages of measurements on three independent cultures and vertical bars show standard deviations (*n* = 3).

Growth of *Synechococcus* sp. PCC7002 in the *Syn* and *Syn*B cultures under different temperatures is shown in Figure 1. The μ_*max*_ of *Synechococcus* in the axenic culture were 0.12 ± 0.02, 0.15 ± 0.04, and 0.17 ± 0.03 d^–1^ under 15, 18, and 21^°^C, while that in *Syn*B culture were 0.09 ± 0.02, 0.18 ± 0.02, and 0.18 ± 0.01 d^–1^, indicating the inhibitory effect of adding bacteria upon the growth merely occurred at low temperature. By contrast, symbiotic heterotrophic bacterial bacteria often promote the growth of algae by providing them with micronutrients like vitamins or bioavailable trace metals (Amin et al., 2009). Such a positive effect is closely related to temperature according to our results. Although adding bacteria showed a limited effect on the growth of *Synechococcus* at higher temperatures, it indeed induced approximately 50% promotion in cell density at both 18 and 21^°^C at stationary phase if comparing the cell abundance of *Syn* culture to that of *Syn*B culture (i.e., 1.21 × 10^9^ ± 2.02 × 10^7^ vs. 2.67 × 10^9^ ± 5.52 × 10^7^ at 18^°^C; and 1.26 × 10^9^ ± 1.46 × 10^7^ vs. 2.80 × 10^9^ ± 2.13 × 10^7^ at 21^°^C). Consistent with this finding, a model result by Flombaum et al. (2013) showed that the abundance of *Synechococcus* increases in the future oceans. It was worth noting that the final cell density of *Synechococcus* at 15^°^C was insignificant between *Syn* and *Syn*B cultures. This may be explained that the heterotrophic bacteria may have activated the growth of *Synechococcus* through providing them essential micronutrients (Hayashi et al., 2011; Kazamia et al., 2012), but such an activated function just started when temperature is over a threshold of e.g., higher than 18^°^C (Figure 1).

### Common Chromophoric Dissolved Organic Matter Properties

**FIGURE 2:**
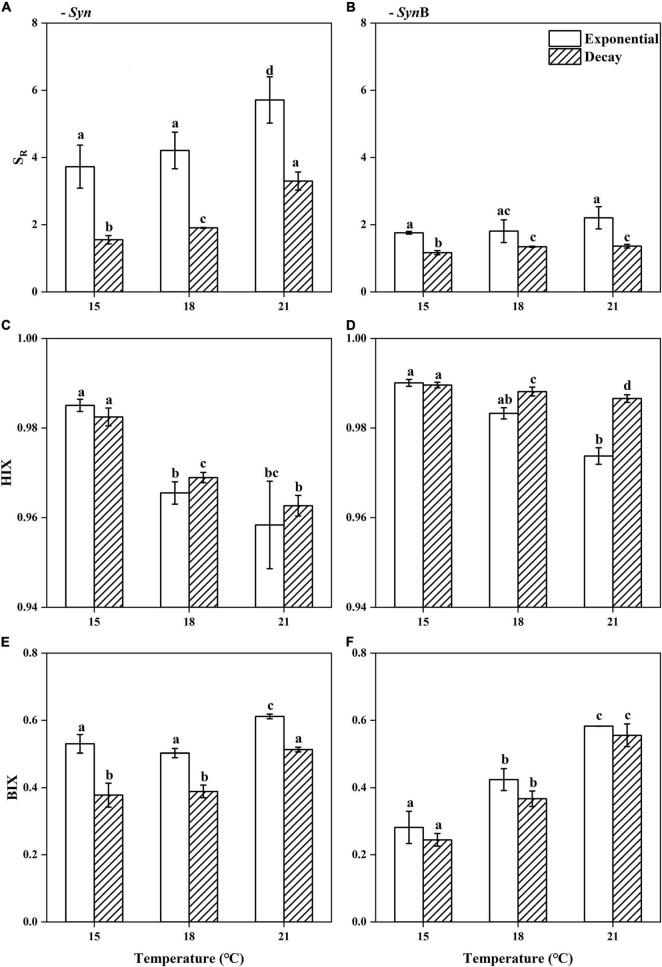
Spectral slope ratio [**(A,B)**, S_*R*_], humification index [**(C,D)**, HIX], and biological index [**(E,F)**, BIX] of *Synechococcus*-derived dissolved organic matter (SOM) at exponential and decay phases, in *Synechococcus* sp. PCC7002 cultures without [**(A,C,E)**, *Syn*] and with heterotrophic bacteria [**(B,D,F)**, *Syn*B] under temperatures of 15, 18, and 21^°^C. Vertical bars indicate standard deviations (*n* = 3), and different letters on top of bars indicate significant difference (paired *t*-test, *p* < 0.05).

Chromophoric dissolved organic matter is an important component of DOM and its vital role in the marine carbon cycle has been widely acknowledged (Yamashita et al., 2010; Andrew et al., 2013). To explore the impacts of temperature on the common CDOM properties of SOM, we measured the absorbance (S_*R*_) and fluorescence indicators (HIX and BIX) of the *Syn* and *Syn*B cultures under three different temperatures (Figure 2), which are well-known to closely correlate with the bioavailability of DOM (Cory and McKnight, 2005; Helms et al., 2008; Huguet et al., 2009; Yuan et al., 2017).

In axenic *Syn* culture, the S_*R*_ of SOM increased from 3.73 ± 0.64 at 15^°^C to 5.71 ± 0.69 at 21^°^C at exponential growth phase, while it increased from 1.55 ± 0.12 to 3.30 ± 0.27 at decay phase (Figure 2A). Higher S_*R*_ values are generally associated with the lower DOM molecular weight and aromaticity (Helms et al., 2008), while the DOM with lower aromaticity is often less stable and more likely to be utilized by heterotrophic microorganisms (Zhou et al., 2020). Therefore, the elevated temperature may have reduced the molecular weight and aromaticity, leading to the better bioavailability of SOM. Moreover, the S_*R*_ value at exponential phase was significantly higher than that at decay phase, no matter under what temperatures (Paired *t*-test, 15^°^C, *t* = 6.94, *p* < 0.05; 18^°^C, *t* = 8.82, *p* < 0.05; 21^°^C, *t* = 4.38, *p* < 0.05), indicating the bioavailability of SOM is higher in former than latter growth phases. A possible explanation is that the more permeability of cell membrane at decay phase allowed the release of intracellular matters like pigments to surroundings, while these metabolites may contribute to the humic-like fluorescent groups (Zhao et al., 2017). It may thus be speculated that the addition of such a humic substance results in the decrease of the bioavailability of SOM. Evidence that elevated temperature enhanced the bioavailability may also be inferred from the HIX and BIX values of SOM, which decreased from 0.99 ± 0.00 to 0.96 ± 0.01 and increased from 0.53 ± 0.03 to 0.61 ± 0.01 with temperature increasing from 15 to 21^°^C at exponential phase (Figures 2C,E). At decay phase, the HIX was similar to that at exponential phase, but the BIX was significantly lower (paired *t*-test, 15^°^C, *t* = 4.61, *p* < 0.05; 18^°^C, *t* = 6.98, *p* < 0.05; 21^°^C, *t* = 13.48, *p* < 0.05), and both HIX and BIX presented similar temperature-dependent changes. HIX values correspond to the ratio of fluorescence intensity between humic and protein-like components at long excitation wavelengths (Ohno, and Tsutomu., 2002), and higher HIX values thus indicate the presence of complex molecules like high molecular weight aromatics (Senesi et al., 1991). It is generally believed that the DOM that can persist for a long time in deep sea often contains large amounts of humic substances that are not easily utilized by microorganisms (Zhao et al., 2017); in contrast, protein-like components have a rapid turnover rate and cannot retain for a long time in marine environment (Zhang and Wang, 2017). The BIX characteristics of autochthonous biological activity in water (Huguet et al., 2009) essentially reflects the ratio of fluorescence intensity of humic-like component peaks at short wavelengths to that at long wavelengths. The fluorescence emission spectra usually move toward longer wavelengths with increasing aromaticity (Senesi, 1990). Therefore, the accumulation of more fluorescent substances at short wavelengths means the higher BIX values and the DOM being more easily utilized; the higher BIX values, the more fresh and higher bioavailability of DOM (Cory and McKnight, 2005; Huguet et al., 2009). Given the lower HIX and higher BIX at 21^°^C condition, the SOM from the cultures under higher temperature have a lower degree of humification and can be retained for a shorter time period. Again, these SOM were inferred from higher bioavailability. The mechanisms underlying this phenomenon need to be studied further. Moreover, consistent with S_*R*_, the lower BIX at exponential than decay phases (Figures 2A,E) also indicated the higher bioavailability of SOM in former than latter phases.

All of the above results appeared to suggest that higher temperature favors higher bioavailability of SOM. Does the heterotrophic bacterial biological process change this? As compared to axenic *Syn* culture, the S_*R*_ of SOM of non-axenic *Syn*B culture was significantly lower among all temperatures in both exponential and decay growth phases (Paired *t*-test, *t* = 5.69, *p* < 0.05), and with an insignificant increase of increasing temperature (Figure 2B). Similar to S_*R*_, the decrease effect by adding heterotrophic bacteria also occurred in the BIX (Figure 2F). The heterotrophic bacteria addition significantly enhanced the BIX at 18 and 21^°^C, but not at 15^°^C (Figure 2F), indicating the modification of heterotrophic bacteria on bioactivity of SOM. Consistently, previous studies demonstrated that heterotrophic bacterial processes can make the DOM more resistant to further degradation (Liu et al., 2021). According to Ogawa et al. (2001),Jiao et al. (2010),Jiao et al. (2011), and Benner (2011), most of the SOM modified by heterotrophic bacteria were converted into inorganic carbon and returned to environments, and the others were converted into more complex organic structures and retained in the environments for the extended time-periods, which was also illustrated in this study.

### Fluorescent Components of *Synechococcus*-Derived Dissolved Organic Matter

During calculation process of the above fluorescence parameters, the limited data such as a single point fluorescence signal (e.g., BIX) or the segment of fluorescence signal at a single excitation wavelength (e.g., HIX) were considered, which inevitably introduced into the deviations. The PARAFAC component analysis was thus performed to validate the changes of the bioavailability of SOM. To characterize the structures of SOM, we detailed its fluorescence and identified a total of five main components (C1–C5) from all samples according to EEM-PARAFAC model (Supplementary Figure 1), being detailed in Table 1. Components 1–3 and 5 have been evidenced as humus-like substances (Stedmon et al., 2003; Stedmon and Markager, 2005a,b; Murphy et al., 2008; Yamashita et al., 2008; Zhang et al., 2009), and the Component 4 as protein-like substances (Yu et al., 2015). Components 1–3 are usually considered as terrestrially derived humic substances (Coble, 1996); while some studies have also identified these components as algae-derived DOM (Mou et al., 2017; Zhao et al., 2017), also in this study (Supplementary Figure 1). Component 5 has also been reported to be associated with heterotrophic microbial processes (Zhang et al., 2009). But we found it not only presented in *Syn*B culture, but also in axenic culture of *Synechococcus*; it suggested the participation of heterotrophic bacteria was not necessary to produce Component 5. Furthermore, component 1 displayed similar optical properties as the fluorescent component BATS1 from deep sea [ex| em: 250 (340)| 460 nm], while the component 5 was similar to component BATS2 [ex| em: 250 (310)| 400 nm] (Zhao et al., 2017). Our results again confirmed the conclusion that *Synechococcus* is one of an important source of deep-sea autochthonous FDOM (Zhao et al., 2017; Zheng et al., 2020). Previous studies have also identified the protein-like components including tyrosine- and tryptophan-like fluorophores (Stedmon and Markager, 2005a,b), and Component 4 in this study could be identified as autochthonous tyrosine-like fluorophores (Yu et al., 2015). In general, it is speculated that all the fluorescent components identified in this study could be produced by *Synechococcus* that may do the contribution in field oceans.

**TABLE 1:** Fluorescent components identified by parallel factor analysis (PARAFAC).

Components	Ex/Em maximum wavelength (nm)	Descriptions	References
C1	250/455	Humic-like substances	Stedmon et al., 2003;
C2	260 (360)/450		Stedmon and Markager, 2005a,b;
C3	250 (380)/480		Murphy et al., 2008;
C5	330(< 250)/400		Yamashita et al., 2008; Zhang et al., 2009
C4	275/300	Tyrosine-like fluorescence	Yu et al., 2015

*The secondary peaks are shown in parentheses.*

**FIGURE 3:**
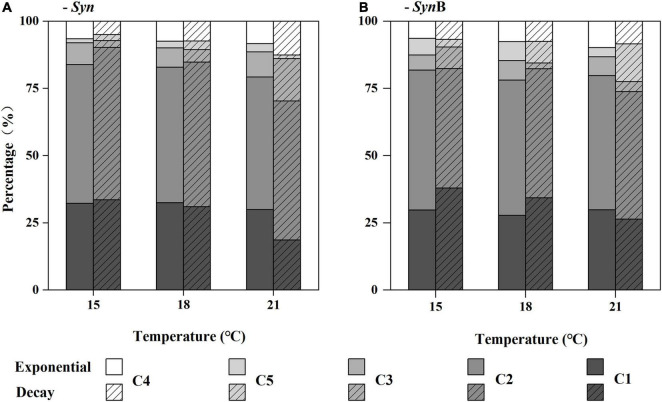
Relative contribution (%) of the main fluorescence components (C1, C2, C3, C4, and C5) to total chromophoric dissolved organic matter (CDOM) fluorescence (Raman Units, RU) of *Synechococcus*-derived dissolved organic matter (SOM) at exponential and decay phases, in *Synechococcus* sp. PCC7002 cultures without [**(A)**, *Syn*] and with heterotrophic bacteria [**(B)**, *Syn*B] under temperatures of 15, 18, and 21^°^C.

Difference in contribution of the five components to total CDOM fluorescence intensity in each DOM sample is shown in Figure 3 (the data quality as indicated by the SD versus mean value of three biological replicates is shown in Supplementary Figure 2). In general, the humus-like fluorescent substances (C1–3 and C5) are dominant in SOM (i.e., 87–95%). A similar result was also reported by Zheng et al. (2020). Furthermore, the humus-like fluorescent components derived from both *Syn* and *Syn*B cultures were mainly the components 1 and 2 (over 70%). Similar results have also been found in some other *Synechococcus* cultures, such as *Synechococcus* CB0101 and *Synechococcus* sp. YX04-3 (Zhao et al., 2017; Zheng et al., 2019). In *Syn* culture, the sum of humus-like substances (C1, C2, C3, and C5) significantly decreased with increasing temperature in both exponential (R^2^ = 0.99, *p* < 0.05) and decay growth phases (R^2^ = 0.97, *p* < 0.05), although no clear trend was observed in each component (Figure 3A). In protein-like component (C4), however, this trend contrarily increased. The protein-like component has simpler cellular structure and could be utilized more easily by heterotrophic bacteria, as compared with the humus-like component (Zheng et al., 2020). Therefore, the increased protein-like component could be responsible for the increase of the bioavailability of SOM at elevated temperature. In addition, reliability of the results about the changes of SOM bioavailability from optical indicators was also considered. However, based on the FDOM data, the reasons why the higher bioavailability of SOM occurred in exponential growth phase and was not modified by heterotrophic bacteria could not be explained at present (Figure 3); this would be further explained by the molecular composition of the SOM identified with FT-ICR-MS.

### Chemical Composition of *Synechococcus*-Derived Dissolved Organic Matter

**FIGURE 4:**
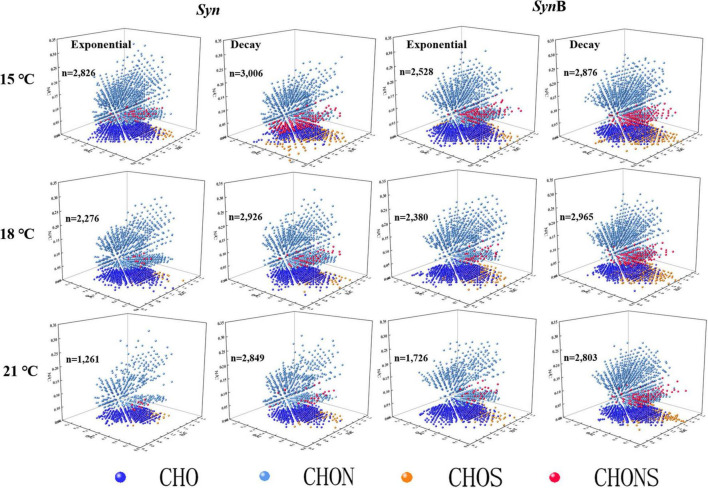
3D Van Krevelen diagram showing the characteristics of each molecule identified by Fourier transform ion cyclotron resonance mass spectrometry (FT-ICR MS). Each ball represents a molecular formula and “*n*” is the total number of molecular formulae. Dark blue, light blue, orange, and red colors indicate the formulae containing element CHO, CHON, CHOS, and CHONS, respectively.

The FT-ICR-MS analysis can provide information about the molecular compositions of DOM (Martínez-Pérez et al., 2017; Zark and Dittmar, 2018) that may indicate its bioavailability through revealing the large diversity of organic compounds (Figure 4 and Supplementary Figure 3). Due to the limitation of extraction efficiency of PPL cartridge, here we just analyzed the extractable and ionizable components of DOM. The 3D Van Krevelen diagram showed the detailed information of SOM molecules in terms of element compositions, i.e., oxygen to carbon ratio (O/C), H/C and nitrogen to carbon ratio (N/C) (Figure 4). Based on the element compositions (CHO, CHNO, CHOS, and CHNOS), the SOM can be classified into four groups. Apart from C, H, and O, they also contained abundant nitrogen and sulfur, consistent with previous studies that reported the DOM released by marine *Synechococcus* was a substantial source of *in situ* nitrogen- and sulfur-containing compounds (Zhao et al., 2017; Zheng et al., 2019, 2020). In *Syn* culture at 15°C temperature, 2,826 and 2,528 molecular formulas were identified in SOM in exponential and decay growth phases, and the numbers were 2,276 and 2,380 at 18°C, and 1,261 and 1,726 at 21°C, respectively (Supplementary Figure 3). The types of identified molecular formulas of SOM were more simplified at higher temperatures, indicating the ocean warming may make the SOM composition more and more simplification. Moreover, the bacteria addition has increased the diversity of SOM molecules (Supplementary Figure 3), suggesting the associated heterotrophic bacteria have contrarily changed this phenomenon.

**FIGURE 5:**
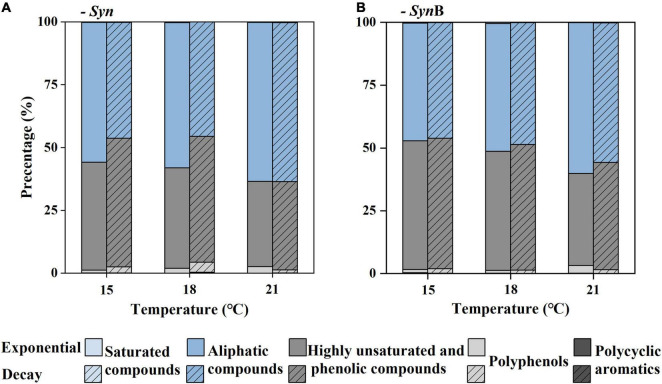
Relative abundance (%) of classification classes based on the modified aromatic index (AImod) values and hydrogen to carbon ratio (H/C) of *Synechococcus*-derived dissolved organic matter (SOM) at exponential and decay phases, in *Synechococcus* sp. PCC7002 cultures without [**(A)**, *Syn*] and with heterotrophic bacteria [**(B)**, *Syn*B] under temperatures of 15, 18, and 21^°^C. The polycyclic aromatics are defined as AImod > 0.66, polyphenols as 0.5 < AImod < 0.66, highly unsaturated and phenolic compounds as AImod ≤ 0.5 and H/C < 1.5, aliphatic compounds as 1.5 ≤ H/C < 2, and saturated compounds as AImod < 0.5 and H/C > 2.

To further explore the compositions of SOM, the DOM compounds were divided into five classifications according to AImod value and H/C ratio, among which the sum of highly unsaturated and phenolic compounds and aliphatic compounds accounted for more than 95%, while the remaining (i.e., sum of polycyclic aromatics, polyphenols, and saturated compounds) accounted for less than 5% (Figure 5). According to Liu and co-authors the algal-derived DOM are lack of compounds with the AImod > 0.67 (Liu et al., 2021). Our study further indicated that the proportion of compounds with AImod > 0.5 (polycyclic aromatics and polyphenols) was low in SOM. The saturated compounds were shown with low abundance in various DOM samples (Seidel et al., 2014, 2015; Qiao et al., 2020), consistent with this study. As below, we focused on two more abundant compounds (i.e., highly unsaturated and phenolic compounds and aliphatic compounds). The proportion of aliphatic compounds in *Syn* cultures increased from 46.14 to 63.24% as temperature increased from 15 to 21^°^C at exponential growth phase. At decay phase, a similar temperature-dependent increase trend occurred. In contrast, the proportion of highly unsaturated and phenolic compounds decreased with increasing temperature. The modification of cellular fatty acid composition has been widely reported to maintain the normal cell growth across temperatures (Thompson, 1996). Compared with highly unsaturated and phenolic compounds, the aliphatic compounds had higher biodegradability and more rapidly turnover rate (Kellerman et al., 2018). Therefore, it is likely that the increase of aliphatic compounds in SOM caused by elevated temperature was responsible for the increase of its bioavailability. Moreover, the proportion of aliphatic compounds was 1–12% higher in exponential than decay phases among all temperature treatments (Figure 5). It may also explain why the bioavailability of SOM in the exponential growth phase was relatively high due to the lower proportion of highly unsaturated and phenolic compounds and higher aliphatic compounds. This result is consistent with the findings of Liu et al. (2021) that the amount of more recalcitrant compounds increased with the growth and degradation of algal cells. Most of SOM in exponential growth phase was secreted by *Synechococcus* cells, while in decay growth phase it was a mixture with intracellular substances exuded by cell death. It could be speculated that the bioavailability of intracellular SOM was lower than that being secreted during cell growth. In *Syn*B cultures, the proportion of highly unsaturated and phenolic compounds in culture *Syn*B was about 3–9% higher than that in culture *Syn* in both growth phases (Figure 5). It is possible that heterotrophic bacterial modifications play an important role in formation of highly unsaturated and phenolic compounds. The heterotrophic bacteria usually prefer to utilize the relatively well-used aliphatic compounds rather than highly unsaturated and phenolic compounds, during which part of aliphatic compounds were transformed into inorganic matters by respiration and the remaining may be modified to other components and retained in cultures (Jiao et al., 2010; Hach et al., 2020). So, the proportion of highly unsaturated and phenolic compounds was higher and that of aliphatic compounds lower in *Syn*B cultures, thus suggesting that heterotrophic bacteria modify the bioavailability of SOM by altering their molecular composition.

Overlap and difference of molecular formulas of SOM among different treatments are illustrated in Supplementary Figure 4: the similarities and differences among different growth phases or cultures without or with bacteria (Supplementary Figure 4A) and among different growth temperatures (Supplementary Figure 4B). Approximately two-thirds of molecular formulas of SOM in different cultures or growth phases were shared, and no unique part was observed, while they varied greatly with temperature changes. So, we pooled all the SOM at each temperature together and found the molecular formulas that were shared by three growth temperatures accounted for 54.56% (Supplementary Figure 4B), indicating these molecules were insensitive to temperature changes. The temperature-sensitive molecules accounted for 13.71, 9.78, and 3.59% at 15, 18, and 21°C, respectively, consistent with the result that the most identified molecular formulas presented at 15°C.

**FIGURE 6:**
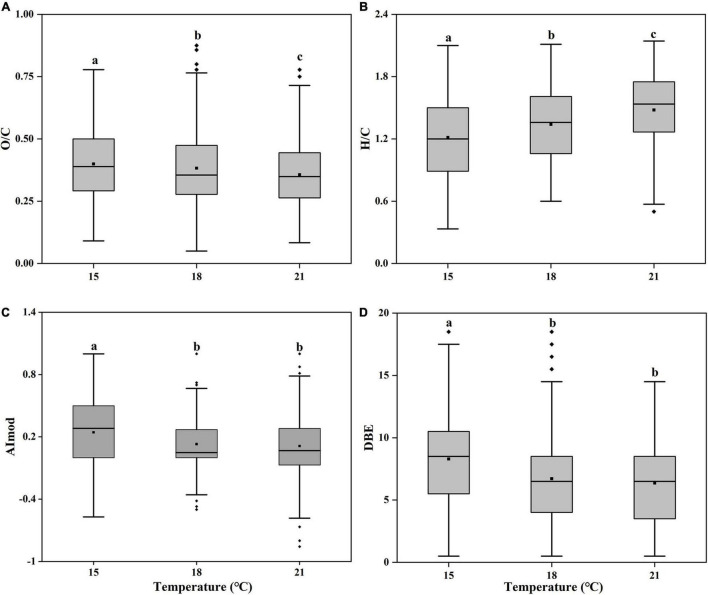
Box plots of pooled oxygen to carbon ratio [**(A)**, O/C], hydrogen to carbon ratio [**(B)**, H/C], modified aromatic index [**(C)**, AImod] and double bond equivalents [**(D)**, DBE] of unique molecular formulas of the *Synechococcus*-derived dissolved organic matter (SOM) of *Synechococcus* sp. PCC7002 cultures without and with heterotrophic bacteria at both exponential and decay phases under temperatures of 15, 18, and 21^°^C. Bottom and top of the box correspond to 25th and 75th percentiles, and the line in box shows the median. Black squares represent the mean values. Vertical lines outside the box represent 10th and 90th percentiles, and filled diamonds denote the outliers. Different letters represent significant differences (paired *t*-test, *p* < 0.05).

By further analyzing the O/C, H/C, AImod, and DBE of these unique molecular formulas, we found these molecular formulas tended to have higher H/C and lower O/C, AImod, and DBE values at higher temperatures (Figure 6). The averaged O/C values for specific molecules were 0.40, 0.39, and 0.36 at 15, 18, and 21^°^C, while the averaged H/C values were 1.21, 1.34, and 1.48, respectively. Such a decrease in O-containing compounds and increase in H-containing compounds suggested that the bioavailability of organic matters increased and became more well-used by heterotrophic bacteria with increasing temperature (Ohno et al., 2014; Qiao et al., 2020). Moreover, the lower aromaticity in formulas of specific molecules that was higher at higher temperatures (6.71 at 18^°^C, 6.37 at 21^°^C) than lower temperatures (8.30 at 15^°^C) also supported the bioavailability increased with increasing temperatures (Zheng et al., 2019). It was reported that cell membranes typically incorporate more unsaturated compounds at lower temperature, but more saturated substances at higher temperature (Hilditch, 1949; Jacobson et al., 1959). In this study, we confirmed that elevated temperature resulted in the more saturated SOM (Figure 6D). The averaged DBE value decreased from 0.24 to 0.11 from 15 to 21^°^C. The low value of DBE represents the more saturated structure of molecules and could be considered as an indicative of the substances with high bioavailability (Koch and Dittmar, 2006). In general, SOM at elevated temperature were H-richer, with a higher aromaticity and relatively saturated (lower DBE value), indicating higher bioavailability.

## Conclusion

Heterotrophic bacteria are of great significance to the growth of marine *Synechococcus* (Zheng et al., 2017); therefore, there have been few studies focused on the SOM that is not modified by bacteria so far. Our study filled this gap and found that the bioavailability of SOM significantly increased with increasing temperatures. Furthermore, through analyzing photochemical properties and molecular compositions, we found the possible reasons for this bioavailability increase of SOM are as follows: (1) the increase of protein-like components; (2) the increase of relatively well-used aliphatic compounds; (3) the increase of hydrogen-containing molecules; and decrease of the unsaturation and aromaticity degrees. In addition, the heterotrophic bacterial processes and intracellular DOM release may decrease the bioavailability, but it did not change the increased trend of the SOM bioavailability with increasing temperatures. Considering the wide distribution of *Synechococcus* and its abundance increase with increasing temperature (Morán et al., 2010), our results gave a perspective that warming may enhance the bioavailability of certain SOM in global oceans.

## Data Availability Statement

The original contributions presented in the study are included in the article/Supplementary Material, further inquiries can be directed to the corresponding author/s.

## Author Contributions

JL and GL were in charge of experimental guidance and manuscript revision. JZ was in charge of cultivating experiments and manuscript writing. JZ and XC were in charge of data analysis. DL was in charge of maintenance of laboratory equipment and guidance of cultivating experiments. Molecular determination of the samples was performed by CH and QS. All authors designated in this manuscript contributed to the article and approved the submitted version.

## Conflict of Interest

The authors declare that the research was conducted in the absence of any commercial or financial relationships that could be construed as a potential conflict of interest.

## Publisher’s Note

All claims expressed in this article are solely those of the authors and do not necessarily represent those of their affiliated organizations, or those of the publisher, the editors and the reviewers. Any product that may be evaluated in this article, or claim that may be made by its manufacturer, is not guaranteed or endorsed by the publisher.
