# Supplementary material for: Temperature Rise Increases the Bioavailability of Marine Synechococcus-Derived Dissolved Organic Matter
Source: Front Microbiol. 2022 Apr 19;13:838707. doi: 10.3389/fmicb.2022.838707 (PMC9097602; doi:10.3389/fmicb.2022.838707)
Supplement: Supplementary file 1 [file Data_Sheet_1.docx]

Supplementary Materials

**Table S1.** Information of the strains isolated from unpurified *Synechococcus* sp. PCC7002 culture. **#**, the strain used in this study.

| **Class** | **Species** | **Percentage (%)** |  |
| --- | --- | --- | --- |
| *Gammaproteobacteria* | *Halomonas* sp. | 43% | **#** |
| *Gammaproteobacteria* | *Alcanivorax* sp. | 17% |  |
| *Alphaproteobacteria* | *Ponticoccus* sp. | 13% |  |
| *Alphaproteobacteria* | *Nitratireducto* sp. | 13% |  |
| *Chitinophagia* | *Sediminibacterium* sp. | 10% |  |
| *Cytophagia* | *Roseivirga* sp. | 3.3% |  |


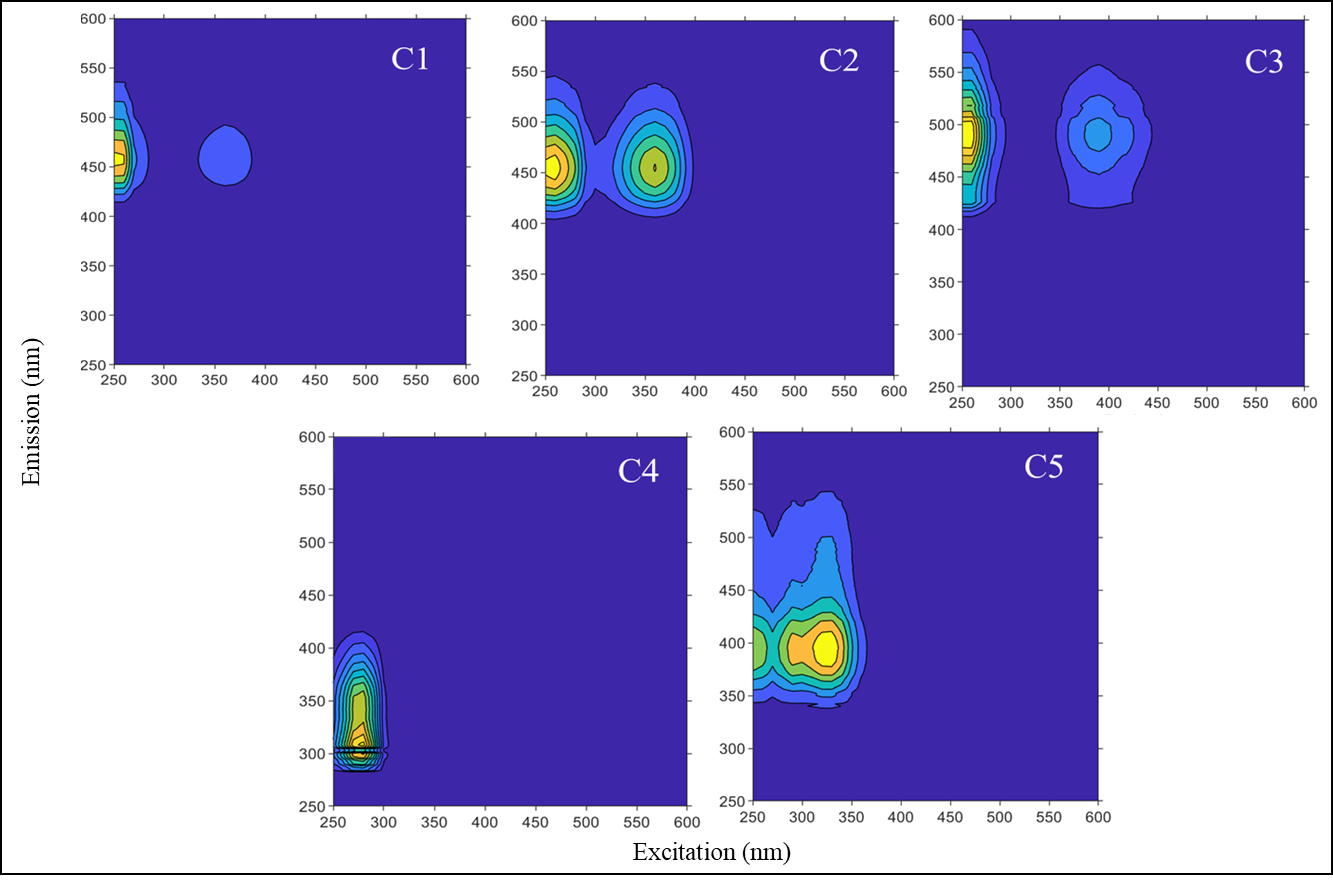


**Supplementary Figure 1.** Contour plots show the fluorescence signatures of 5 components identified by EEM-PARAFAC from all cultures.

**Supplementary Figure 2.** Percentage (%) of standard deviation to mean value plotted against the mean value of three biological replicates of total chromophoric dissolved organic matter (CDOM) fluorescence in Raman Units (RU), to indicate the data quality.


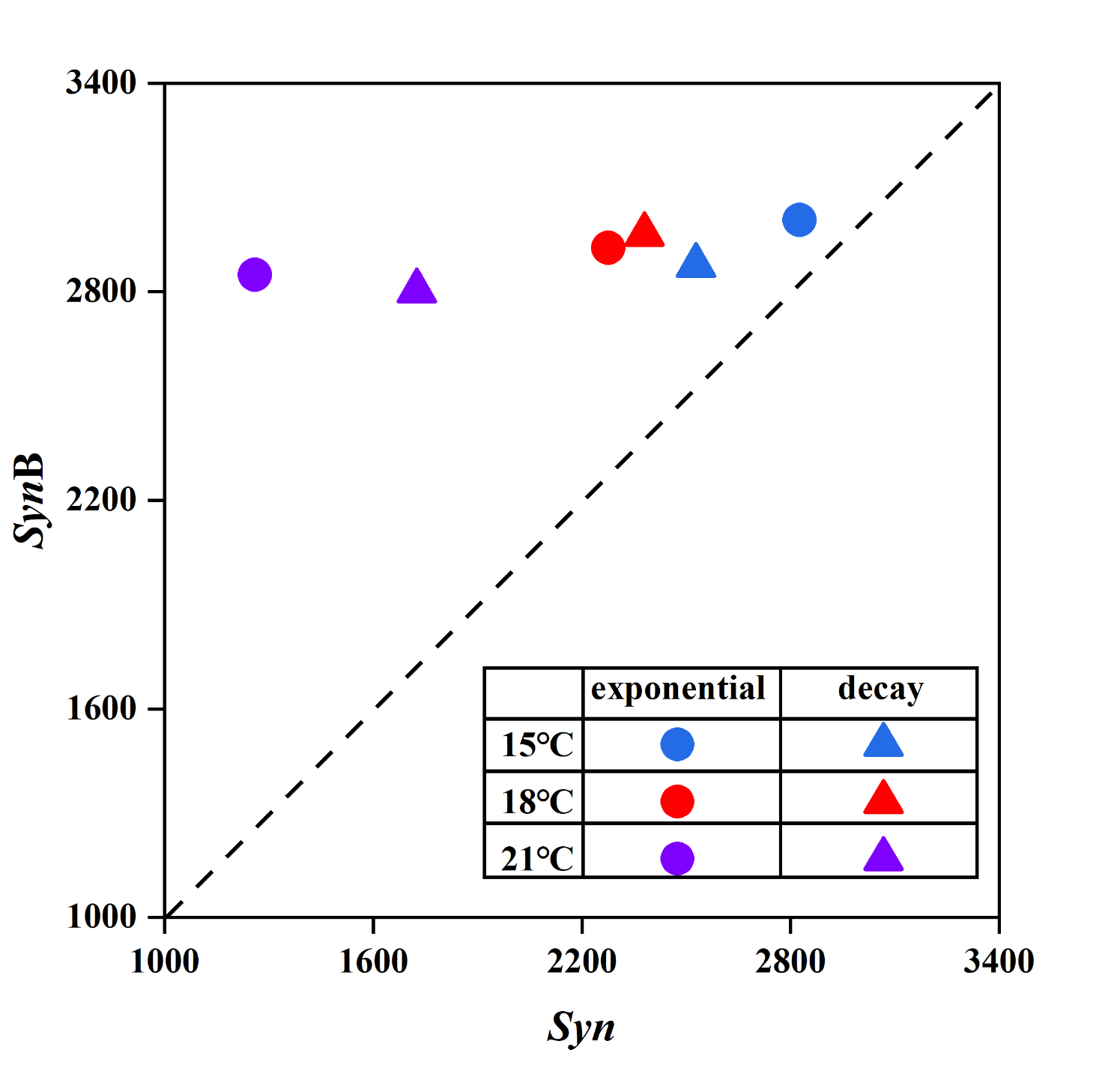


**Supplementary Figure 3.** Molecular formulae number identified from *Synechococcus*­-derived dissolved organic matter (SOM) of *Syn* culture plotted against that of *Syn*B culture at different growth phases and temperatures. Dotted line shows the 1:1 line.


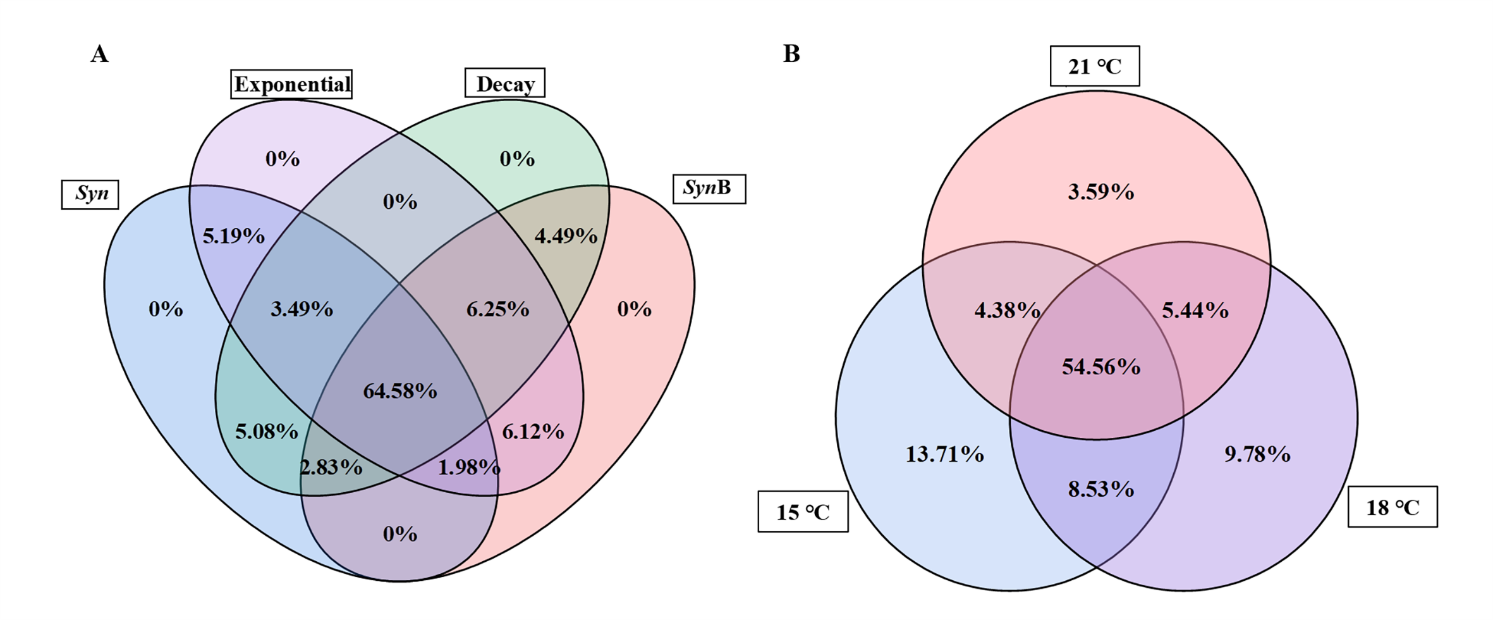


**Supplementary Figure 4.** Venn diagrams showing similarities and differences of the molecular formulas of *Synechococcus*­-derived dissolved organic matter (SOM) among different culture systems or growth phases (A) and among different growth temperatures (B).
